# Suspected Gastroparesis With Concurrent Gastroesophageal Reflux Disease Induced by Low-Dose Liraglutide

**DOI:** 10.7759/cureus.26916

**Published:** 2022-07-16

**Authors:** Yo Ishihara, Sho Nishiguchi, Joel Branch, Eri Tanaka

**Affiliations:** 1 General Internal Medicine, Shonan Kamakura General Hospital, Kanagawa, JPN; 2 General Internal Medicine, Yao Tokushukai General Hospital, Osaka, JPN; 3 Internal Medicine, Hayama Heart Center, Kanagawa, JPN

**Keywords:** type 2 diabetes mellitus, adverse side effect, glp-1 agonist, reflux esophagitis, gastroparesis treatment

## Abstract

A 74-year-old woman with type 2 diabetes mellitus presented with nausea and abdomen distension. Four days prior, liraglutide 0.6 mg had been commenced. An abdominal computed tomography scan revealed gastric dilatation without mechanical obstruction which clinically suggested gastroparesis (GP). Her symptoms resolved after liraglutide discontinuation. A gastroscopy revealed reflux esophagitis. Taken together, GP may have developed along with reflux esophagitis due to liraglutide administration. Liraglutide’s action inhibits gastric motility. Physicians should be cognizant of the side effects of GLP-1 agonists even in low dose in patients who have gastric emptying symptoms suggesting GP.

## Introduction

Gastroparesis (GP) is a condition that is characterized by findings of delayed gastric emptying without mechanical obstruction of the pylorus or duodenum [1]. GP causes gastrointestinal symptoms such as abdominal distention, nausea, vomiting, and gastric acid reflux.

Causes of GP include diabetes mellitus (DM), thyroid dysfunction, neurological disease, prior gastric or bariatric surgery, autoimmune disorders, and drugs such as glucagon-like peptide type 1 (GLP-1) analogs [1,2]. Liraglutide, one of the GLP-1 analogs used for the treatment of DM, is a drug that stimulates insulin secretion from pancreatic beta-cells, inhibits glucagon secretion from pancreatic alpha cells, and suppresses gastric peristalsis [3,4]. Liraglutide not only lowers blood glucose in DM patients but also enhances pancreatic beta cell function, decreases body weight, and exhibits antihypertensive effects when administered once daily. Often in a dose-dependent manner, liraglutide has side effects including nausea and abdominal distension [3]. It is also reported to cause decreased esophageal peristalsis [5]. However, there have been only a few case reports citing GP induced by liraglutide [2,4]. Here, we report a case of suspected GP in a patient with type 2 DM (T2DM) newly treated with liraglutide.

## Case presentation

A 74-year-old woman visited our emergency room complaining of a sore throat, anorexia, nausea, and abdomen distention which commenced three days prior to admission (PTA). The patient had a history of hypertension and type 2 diabetes mellitus (T2DM), the latter was managed with metformin 500 mg twice daily, repaglinide 0.25 mg twice daily, and sitagliptin 50 mg once daily for more than six months. Liraglutide was prescribed four days PTA. She denied a history of cigarette smoking, alcohol, and drug misuse.

Abdominal examination revealed that her abdomen was soft and non-tender but with decreased bowel sounds. Laboratory tests revealed a blood glucose level of 177 mg/dL, hemoglobin A1c of 8.2 %, liver function tests and complete blood counts were within normal limits (Table 1).

**Table 1:** Laboratory tests on admission

Hematology			Chemistry					
White blood cells	10,100	/µL	Aspartate aminotransferase	26	U/L	Uric acid	6.2	mg/dL
Lymphocyte	24.9	%	Alanine aminotransferase	20	U/L	Total cholesterol	170	mg/dL
Neutrophil	69.5	%	Lactate dehydrogenase	191	U/L	Triglyceride	116	mg/dL
Eosinophil	0.7	%	γ-Glutamyl transpeptidase	14	IU/L	High-density lipoprotein	46.5	mg/dL
Basophil	0.3	%	Amylase	66	IU/L	Sodium	139	mmol/L
Red blood cells	410	×10^4^/µL	Total protein	7.9	g/dL	Potassium	4.7	mmol/L
Hemoglobin	12.3	g/dL	Serum albumin	4.6	g/dL	Chlorine	100	mmol/L
Hematocrit	36.9	%	Blood urea nitrogen	20.5	mg/dL	Calcium	9.7	mg/dL
Mean corpuscular volume	90	fL	Serum creatinine	0.97	mg/dL	C-reactive protein	0.02	mg/dL
Mean corpuscular hemoglobin	30	pg	Estimated glomerular filtration rate	43.1	mL/min/1.73 m^2^	Thyroid-stimulating hormone	2.7	µIU/mL
Mean corpuscular hemoglobin concentration	33.3	%	Fasting glucose	177	mg/dL	Free thyroxine 4	1.3	ng/dL
Platelets	27	×10^4^/µL	HemoglobinA1c	8.2	%	Creatine kinase MB	21	IU/L
-	-	-	-	-	-	Troponin	0.009	ng/mL

**Figure 1:**
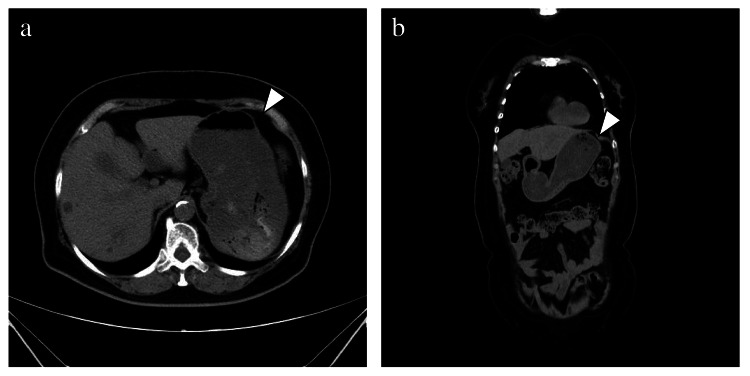
Abdominal computed tomography scan Axial (a) and coronal (b) sections of abdominal CT scan on the arrival to the hospital show a dilated stomach (white arrowhead).

A computed tomography (CT) scan of her abdomen revealed fluid accumulation from the stomach to the horizontal portion of the duodenum, but no signs of dilated small or large bowel, or evidence of mechanical obstruction (Figures 1a, 1b). In view of severe nausea, a nasogastric tube was inserted, which aspirated 600 mL of gastric contents. Intravenous metoclopramide 10 mg was also administered.

**Figure 2:**
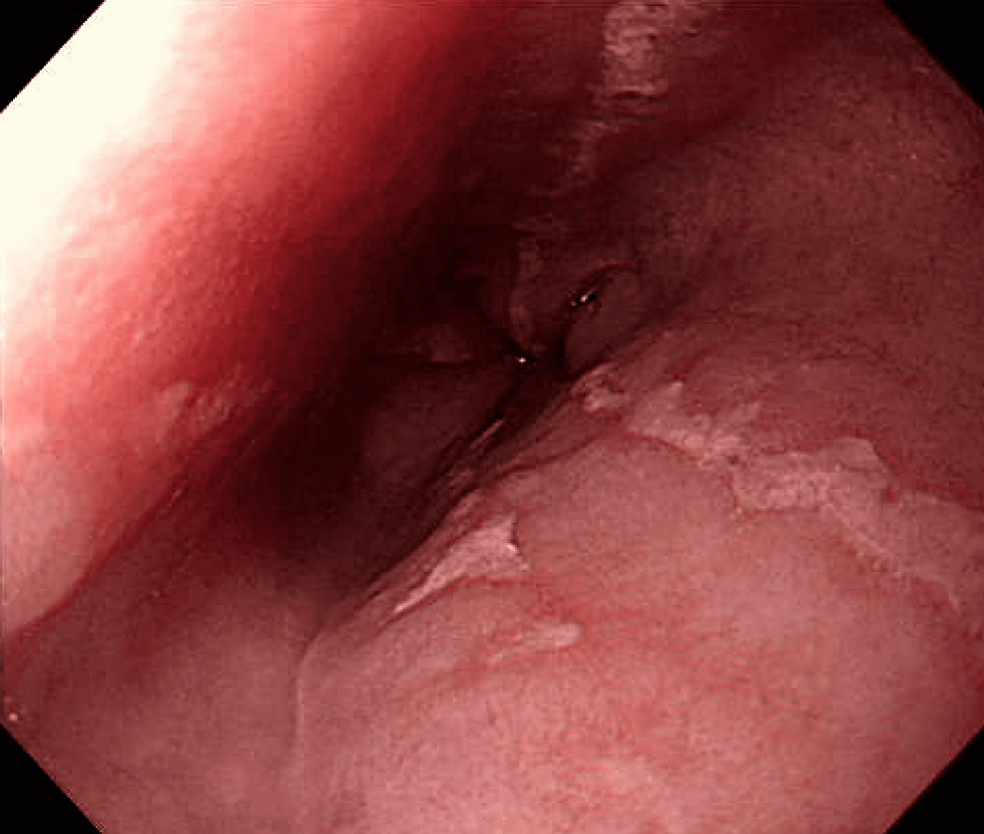
Photograph of the gastroesophageal junction Gastroscopy showing gastroesophageal junction with findings of Los Angeles grade C reflux esophagitis.

The patient was admitted to the ward. Oral nutrition and all diabetic medications were discontinued. Her diabetes was managed with rapid-acting insulin from the day of admission. A gastroscopy was performed to investigate the cause of nausea, which showed a patent pylorus along with reflux esophagitis (RE) of Los Angeles grade C (Figure 2). On the third day of admission, nausea resolved and the patient was able to take oral nutrition.

Since there was a history of the initiation of liraglutide before admission, we suspected that it may have induced GP. Diabetes treatment was then changed from liraglutide to insulin degludec 5 units per day. All other anti-diabetic medications, excluding liraglutide, were recommenced. Following this change, the serum glucose level was elevated post-prandially, but fasting glucose was within the normal range. The patient was discharged on the sixth day. By the 10th day, her nausea and reflux symptoms had entirely disappeared.

## Discussion

We describe a case of a patient with T2DM who complained of nausea, anorexia, sore throat, and abdominal distention. The constellation of symptoms is likely to be due to GLP-1 agonist treatment with liraglutide which caused GP and RE.

GP is a syndrome that causes nausea, vomiting, abdominal distention, early satiety, post-prandial fullness, and upper abdominal pain. Causes of GP include poorly controlled DM, post-surgery, viral infections, parkinsonism, idiopathic, amyloidosis, paraneoplastic syndrome, scleroderma, mesenteric ischemia, and drugs such as narcotics, anticholinergics, and GLP-1 analogs [1][6]. The pathogenesis of GP is complicated and is thought to involve the interstitial cells of Cajal which mediate communication between the autonomic nervous system and the smooth muscle of the alimentary tract. This is mediated via neuronal nitric oxide synthase. The diagnosis of GP requires the demonstration of delayed gastric emptying through several methods including scintigraphy, which is the gold standard test, wireless motility camera, or carbon breath testing. In addition, mechanical obstruction must be excluded [1,4]. In this case, we could not perform examination for delayed gastric emptying because of limited resources in our semi-rural community hospital. However, the aforementioned clinical symptoms and imaging suggest that GP was the most likely clinical diagnosis.

GP due to liraglutide is a rarely reported but major side effect. Thus drug-induced GP should be considered in T2DM patients [4,6]. There are case reports of liraglutide-induced GP having occurred with a dose of 1.2 mg in a 52-year-old male patient [4] and with a dose of 3 mg in an 18-year-old female patient [2]. One study compared placebo to 1.8 mg of liraglutide, which demonstrated a 13% reduction in one-hour gastric emptying time in the liraglutide group compared to placebo. This study revealed that even standard dose treatment can cause GP [7]. However, there are no reports exhibiting GP at low doses of liraglutide such as 0.6 mg. Although it is recommended that liraglutide be introduced at a low dose of 0.6 mg once daily and increased over several weeks, this case suggests that GP can possibly occur even at the recommended starting dose [3].

The management of liraglutide-induced GP is largely symptomatic treatment. In addition to the discontinuation of liraglutide, aspiration of gastric contents, prokinetic peristaltic agents such as serotonin and dopamine antagonists, and 5-hydroxytryptamine-4 receptor agonists have been useful for GP [1,8]. Persistently, high blood glucose levels of 288-360 mg/dL may result in inhibition of gastric emptying of solid and fluid contents [1]. GP itself occurs in about 50% of patients with type 1 and type 2 diabetes mellitus. Hyperglycemia, autonomic neuropathy, inflammation, and damage to the enteric neuromuscular system are thought to contribute to the pathogenesis of GP [9]. Glycemic control is also important in the management of GP.

In the present case, gastroscopy revealed grade C RE (Figure 2). We speculate that reflux esophagitis was the cause of the sore throat [10]. There was no history of RE in this patient’s past. Thus, it is conceivable that RE was caused by liraglutide in this case. Although it is difficult to completely rule out the possibility that RE developed before liraglutide initiation, it seems likely given the chronology of this case. Screening for RE with gastroscopy prior to commencing GLP-1 agonist treatment may be beneficial because such medications are a risk factor for the development and exacerbation of this disorder.

## Conclusions

GP is a known serious side effect of GLP-1 agonist treatment such as liraglutide. Initiation of this class of drug in patients with T2DM may induce GP regardless of the dose administered. GP due to GLP-1 agonist treatment can be ameliorated by (1) discontinuation of the drug, (2) pursuing tighter control of serum glucose with alternative antidiabetic agents, (3) antiemetic agents, and (4) prokinetic agents.

In view that GP can be complicated by RE, the risk of developing or exacerbating this aforementioned disorder should be considered before initiating GLP-1 agonists in general.
